# The Problem with Big Data: Operating on Smaller Datasets to Bridge the Implementation Gap

**DOI:** 10.3389/fpubh.2016.00248

**Published:** 2016-12-01

**Authors:** Richard P. Mann, Faisal Mushtaq, Alan D. White, Gabriel Mata-Cervantes, Tom Pike, Dalton Coker, Stuart Murdoch, Tim Hiles, Clare Smith, David Berridge, Suzanne Hinchliffe, Geoff Hall, Stephen Smye, Richard M. Wilkie, J. Peter A. Lodge, Mark Mon-Williams

**Affiliations:** ^1^School of Mathematics, University of Leeds, Leeds, UK; ^2^Faculty of Medicine and Health, University of Leeds, Leeds, UK; ^3^Big Data and Analytics Unit, Institute of Global Health Innovation, Imperial College London, London, UK; ^4^Leeds Teaching Hospitals NHS Trust, Leeds, UK

**Keywords:** big data, small data, surgery, health economics, length of stay

## Abstract

Big datasets have the potential to revolutionize public health. However, there is a mismatch between the political and scientific optimism surrounding big data and the public’s perception of its benefit. We suggest a systematic and concerted emphasis on developing models derived from smaller datasets to illustrate to the public how big data can produce tangible benefits in the long term. In order to highlight the immediate value of a small data approach, we produced a proof-of-concept model predicting hospital length of stay. The results demonstrate that existing small datasets can be used to create models that generate a reasonable prediction, facilitating health-care delivery. We propose that greater attention (and funding) needs to be directed toward the utilization of existing information resources in parallel with current efforts to create and exploit “big data.”

The “big data” revolution is central to the long-term vision of health services across the globe (1). For example, big data are central to the UK’s Department of Health plans to save £5bn by 2020 through improved operational productivity (2). However, there is a mismatch between the political and scientific optimism surrounding big data and the public’s perception of its benefit (3). In this regard, Big Data constitute a deceptively difficult health-care policy. The research community needs to persuade a skeptical public whose personal health data should be made available for analysis if the big data recommendations are to be realized (4, 5) – see for example, the controversial NHS England “care.data” program. Our concern is that the lack of demonstrable *benefits* from data analytics in the short-term may reinforce skepticism and erode government enthusiasm (and support) for big data projects. The UK, where national policy on Big Data is currently under review (6), might serve as a useful test-bed for other countries. We propose that one of the solutions to the many problems facing Big Data could be bridged by demonstrating the benefits of data analytics using smaller, readily available data.

There are already many examples of local and regional routinely collected data sets being used to improve health-care services (4). In fact, the idea of health service research providing useful information to hospital management is, of course, far from new. These cases suggest that analyzing existing, routinely available health data (“small data”) might be a good starting point for altering public perception, given the difficult strategy of exploiting larger datasets. However, progress in these domains often proceeds in an *ad hoc* manner and success is self-contained. We suggest a systematic and concerted emphasis on developing *models* from these data could illustrate how data science can produce tangible benefits. In order to demonstrate the value of a small data model-based approach, we produced a proof-of-concept model predicting hospital length of stay (LOS).

We chose LOS because the average cost of an excess bed is approximately £273 per day, and the average cost of an elective inpatient stay is £3,366 (7). A model that could predict LOS with some accuracy would mean that fewer operations would be canceled at short notice because of a lack of bed space, thus saving staff and equipment costs, and crucially, provide an improved service for patients.

The current system of bed planning stands as a testament to the remarkable abilities of staff within a hospital – individuals who use extensive insight and knowledge to juggle beds in an environment where both acute and emergency operations can change the requirements on a moment-to-moment basis. The complex, dynamical nature of the hospital is analogous to a weather system and shows similar characteristics (for example, “chaotic” features such as a sensitive dependence on initial conditions). The difference between the weather forecaster and the hospital bed planner lies in the quality of the models they can run to simulate the system of interest. Cognitive science has shown that humans are poor at making decisions under conditions of high uncertainty (8) and tend to prioritize immediate problems over longer-term planning (9, 10), whereas mathematical models can assist in optimizing decision making (11). We therefore studied whether we could utilize existing NHS data to build a simple predictive model as a precursor to one who could help forecast the need for beds following elective surgery (as the scheduling of elective operations in a very large acute NHS Trust, Leeds Teaching Hospitals, has an element of flexibility that offers a degree of control to those running hospitals). We used available data that had been routinely collected by clinicians, health service practitioners, and administrators on an internal system on a daily basis.

For illustrative purposes, we created a model (see Datasheet S2 in Supplementary Material) that could use predictors known *a priori*, and *post hoc* knowledge (e.g., operation time) to provide estimations of LOS for patients undergoing laparoscopic cholecystectomy (LC). We focused on this procedure because it is estimated that 10 and 15% of the adult western population have gallstones (12) – the most common and costly digestive disease (13) – and LC is the preferred treatment option for symptomatic gallstones (14). Due to its prevalence, we reasoned that a predictive model might complement individual intuition and help hospitals plan elective procedures and associated beds in a more efficient manner. This could be beneficial as the costs associated with discharging patients too early can be greater than the initial investment of bed stay and a day-case surgery policy is not suitable across all specialties and procedures – despite demonstrable success in some areas (15). Previous research indicates that modeling LOS is technically feasible (16, 17), yet these approaches are rarely used in practice. This is particularly surprising given the costs associated with sub-optimal bed allocation and the nature of current approaches to scheduling – even the most rudimentary model should provide information of value – that could ultimately translate to economic benefits in the long run.

Our analysis revealed that month, weekday, year, patient age, and operation time were all predictive of LOS using data from 2004 to 2012. Figure 1 shows how each predictor influences LOS, if all other predictors are held constant.

**Figure 1:**
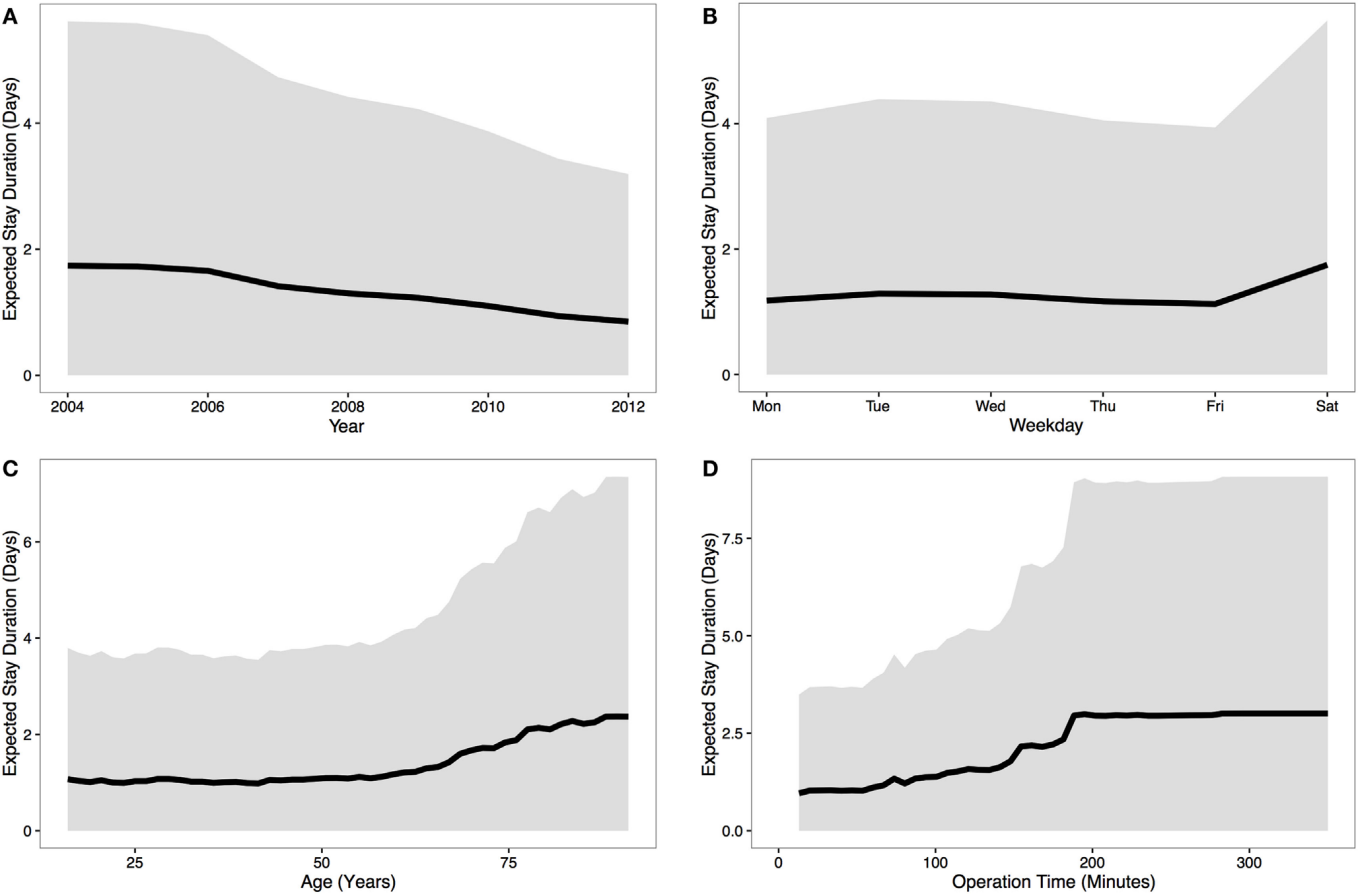
**Predicting patient stay duration**. The results from the model are presented using line plots (the median prediction is represented by a solid black line and the gray region represents the 95% quantile). **(A)** These data show a steady downwards trend in stay duration over the nine relevant years; **(B)** the duration of stay is longer for operations on Saturdays – most likely due to weekday discharge; **(C)** for patients above 55 years of age, stay duration rapidly increases with age; and finally, **(D)** stay duration also increases with operation time – presumably an indicator of complications in surgery or intrinsically more difficult cases. Interestingly, stay duration reaches a plateau for operations over ~3 h, though there are relatively few data points for surgeries of this length – and as such, this relationship should be treated with caution.

Since patient age and operation time were the strongest predictors of stay duration (Figures 1C,D), we extracted a two-dimensional plot of their partial effect in combination (see Figure 2). Note that operation time is a variable only known post-surgery [though surgeon expertise and difficulty are correlates (18)]. The pseudo-*R*^2^ for this model was 0.29. Based on 1,000 permutations where stay durations were permuted and the model refitted, this model reached statistical significance at a threshold of *p* < 0.001. For younger patients (<55 years), we found a relatively weak relationship between operation length and LOS up to the 3-h mark, but for older patients (>55 years), stay duration increased strongly with surgery length.

**Figure 2:**
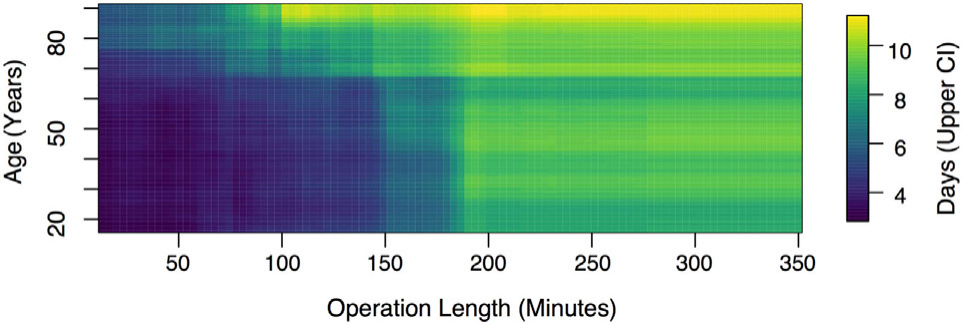
**Upper 95% confidence interval of predicted stay duration**. This figure shows that while, for younger patients, the effect of surgery length on length of stay is relatively weak up to the 3-h mark, for older patients (>55 years), stay duration increases strongly with duration length across all time scales.

There is, of course, substantial between-patient variation unaccounted for by our model (given data limitations), and there is considerable room for further predictive improvement. Intrinsic (but unrecorded) differences between patients are always likely to make prediction difficult. In reality, decision makers face scheduling problems on different time horizons; here, we included operation length as a predictor, but this is clearly not known until the operation is complete. Without this predictor, the best-fit model selects month, year, and patient age as factors, with a pseudo-*R*^2^ value of 0.17. It is remarkable that a simple five-factor model can account for this amount of variance in the data given the complexity of the system.

The results of this exercise demonstrate that existing small data sets could be used to create models that allow a reasonable prediction of hospital LOS after surgery. It is notable that the data we used had considerable limitations. For instance, we focused on one procedure with data from one (albeit, large) NHS Trust, and we did not identify the incidence of complications. These issues could be addressed and may have a substantial impact. If this process is replicated across multiple procedures and hospitals, we could be in a better position to plan for 23 h, 5.5-day facilities instead of full in-patient facilities. This information could ultimately influence how hospitals plan and flex their bed-base.

In summary, we have demonstrated a “proof of concept” that a proportion of the variance associated with patient LOS can be predicted from a limited number of factors. Many applications of medical statistics, such as tests for the efficacy of drugs, require careful experimental design to determine causal effects of putative interventions. In contrast, scheduling problems only require accurate prediction given the observable traits of the patient, since no intervention is proposed. This is where predictive modeling from existing small data sets has the lowest barrier to entry. Systematically recording and utilizing more of these data would allow these data to inform the best computational model and allow schedulers to use the model ahead of time when it can be most efficacious. Crucially, these models could be rapidly developed and deployed from existing datasets. Providing, for example, fewer cancelations of elective operations as a result of the effective implementation of a small data LOS model would provide a tangible example of the benefits of data analytics to the public. We suggest that this could provide *one* solution to the reticence of a public who are skeptical about the benefits of their data being collected, particularly if existing datasets can be utilized in novel and clinically beneficial ways.

Finally, while our example is from the UK NHS – an organization that is the largest health-care provider and one of the largest global producers of health data – the resulting predictive model could be used across other health-care systems. Moreover, a demonstration of the usefulness of data analytics in any country can help change the public’s (and clinicians) perception of the value of big data. The UK NHS Hospital Trusts data systems provide an opportune vehicle by which the big data implementation gap can be addressed and, if successful, could serve as a model for others to follow. We therefore propose that greater attention (and funding) needs to be directed toward the utilization of existing data resources, in parallel with current efforts to create and exploit “big data” sets. It is probable that smaller analytical projects yielding efficiency in the short-term (“small data”) will persuade society of the longer-term merits of exploiting data, as well as identify the challenges and opportunities in analytics in a more tractable fashion than is afforded by still-to-be-created big data repositories.

## Author Contributions

ADW collected the data. RPM analyzed the data. FM, RPM, GM-C, TP, DC, SM, TH, CS, DB, GH, SH, SS, RMW, JPAL, and MM-W wrote the paper.

## Conflict of Interest Statement

The authors declare that the research was conducted in the absence of any commercial or financial relationships that could be construed as a potential conflict of interest.
