# Supplementary material for: The Problem with Big Data: Operating on Smaller Datasets to Bridge the Implementation Gap
Source: Front Public Health. 2016 Dec 1;4:248. doi: 10.3389/fpubh.2016.00248 (PMC5130981; doi:10.3389/fpubh.2016.00248)
Supplement: Supplementary file 1 [file data_sheet_1.docx]

## Supplementary Materials 1: Methods

Data were collected from the computer package used by Leeds Teaching Hospital Trust to record details of all operations (the Galaxy Theatre System). We identified 6,391 laparoscopic cholecystectomies (LCs) recorded in a tertiary referral centre between 02/04/2004 and 31/12/2012. A database search identified all adults who underwent an elective LC. LCs were excluded where additional procedures (e.g. on table cholangiogram or hernia repair) might have inflated the recorded operation time (n = 1,494). Incorrectly coded values were identified and excluded (n = 38). Operation duration (LOP in minutes) and length of post-operative hospital stay (LOS in days) were calculated for all remaining cases with usable data. These data were correlated with trust records to identify date of discharge. Length of operation (in minutes) and length of post-operative hospital stay (i.e. from date of surgery to date of discharge) were calculated from these data.

LCs were coded as acute or elective procedures and additional interventions such as on-table-cholangiogram (OTC), adhesionolysis, biopsy, hernia repair were recorded. The database records time into theatre, anaesthetic induction time, time of incision, time of closure, time patient was transferred into recovery and time onto the ward. All adult patients who underwent elective LC only were included in the analysis. Patients who underwent an acute or additional procedure were excluded. Full inclusion/exclusion criteria are shown in Table 1.

**Table S1: Inclusion and exclusion criteria**

| Inclusion | Exclusion |
| --- | --- |
| Elective procedure | Acute procedure |
| >16 years of age at time of surgery | <16 years of age at time of surgery |
|  | Additional procedure. E.g.  OTC  Liver biopsy  Hernia repair (e.g. paraumbilical) |
|  | Conversion to open |
|  | Robotic cholecystectomy |
