# Supplementary material for: The Problem with Big Data: Operating on Smaller Datasets to Bridge the Implementation Gap
Source: Front Public Health. 2016 Dec 1;4:248. doi: 10.3389/fpubh.2016.00248 (PMC5130981; doi:10.3389/fpubh.2016.00248)
Supplement: Supplementary file 2 [file data_sheet_2.docx]

## Supplementary Materials 2: Analysis

We used a Random Forest model (via the randomforest package in R, Liaw & Weiner 2002) to determine which of the recorded factors in the data were useful predictors of the patient stay duration. Random Forests (Breiman 2001) are a highly flexible non-parametric statistical tool for identifying patterns in data without specifying in advance the nature of the relationships between dependent and independent variable. Since the stay durations are zero-bound and highly right skewed, they were first log-transformed to create a new dependent variable: Y = log(stay duration + ½). Then an exhaustive list of possible combinations of predictors was created from: month, weekday, year, patient age, surgeon specialty and operation time, giving 2^6^ = 64 possible models in total. For each combination we fitted a Random Forest to the dependent variable, Y. The randomforest package automatically provides an estimate of predictive accuracy via out-of-bag (OOB) predictions, and we used this measure, in the form of mean square error in Y, as the goodness-of-fit of the model. This mean square error also provides the confidence interval estimations in our predictions.

Our analysis revealed that all of our putative predictors, month, weekday, year, patient age, surgeon specialty and operation time, were predictive of Y, although month and surgeon specialty added very little to the predictive power and are therefore not shown in the main text. Taking the Random Forest fitted from these predictors we extracted the effect of each predictor in isolation by varying the value of this predictor through its range, while holding all other predictor values constant. The resulting model predictions in Figure 1, transformed back in the original dimensions of stay duration in days, show how each predictor influences the expected stay duration, and the upper 95% confidence interval, if all other predictors remain constant.

**Reference**

## Breiman, L. (2001). Random Forests. Machine Learning, 45 (1): 5–32.

Liaw, A., & Wiener, M. (2002). Classification and regression by randomforest. R News 2 (3): 18–22. *URL: http://CRAN. R-project. org/doc/Rnews*.
